# Immunotherapy in biliary tract cancer: reshaping the tumour microenvironment and advancing precision combination strategies

**DOI:** 10.3389/fimmu.2025.1651769

**Published:** 2025-08-08

**Authors:** Jingnan Xue, Longhao Zhang, Kai Zhang, Kai Zhou, Haitao Zhao

**Affiliations:** ^1^ Department of Hepatobiliary Surgery, People’s Hospital of Anshun City, Anshun, Guizhou, China; ^2^ Department of Liver Surgery, Peking Union Medical College Hospital, Chinese Academy of Medical Sciences & Peking Union Medical College, Beijing, China

**Keywords:** biliary tract cancer, molecular mechanisms, immunotherapy, tumour microenvironment, combination therapy precision treatment strategies

## Abstract

Biliary tract cancer, which includes intrahepatic cholangiocarcinoma, extrahepatic cholangiocarcinoma, and gallbladder cancer, presents a significant clinical challenge because of its aggressive nature and limited therapeutic options. Although standard chemotherapy regimens, such as gemcitabine and cisplatin, are used, the prognosis for advanced biliary tract cancer patients remains poor due to the rapid development of resistance. Recently, advancements in immunotherapy, particularly immune checkpoint inhibitors, have shown promise. However, the response rate in patients with biliary tract cancer is still suboptimal primarily because of the highly immunosuppressive tumour microenvironment. This microenvironment includes a complex network of tumour-associated macrophages, regulatory T cells, and myeloid-derived suppressor cells, all of which contribute to immune evasion. In this review, we discuss the molecular mechanisms that drive biliary tract cancer, focusing on genetic alterations and the role of the TME in immune suppression. We also examine current combination strategies that integrate immune checkpoint inhibitors with chemotherapy and targeted therapies, which have demonstrated superior efficacy over monotherapy. Furthermore, we explore emerging therapeutic approaches, such as metabolic modulation, CAR-T-cell therapy, and mRNA vaccines, which are reshaping the treatment landscape. Finally, we highlight the need for personalized treatment strategies and the development of predictive biomarkers to guide therapy selection. Future research should focus on refining these combination therapies, optimizing patient selection, and validating biomarkers to improve clinical outcomes and survival in biliary tract cancer patients.

## 1 Introduction

Biliary tract cancers (BTCs) are malignant tumours that originate from the biliary system, including intrahepatic cholangiocarcinoma (iCCA), extrahepatic cholangiocarcinoma (eCCA), and gallbladder cancer (GBC). Despite its relatively low incidence globally, BTC is associated with a poor prognosis and limited treatment options. The median overall survival of patients with advanced BTC is typically less than one year (1, 2). Although traditional chemotherapy regimens, such as gemcitabine combined with cisplatin (3, 4), offer short-term relief for some patients, their efficacy is limited, and most cancers rapidly develop resistance. Therefore, new therapeutic strategies are urgently needed to improve patient survival and quality of life.

In recent years, immunotherapy, particularly immune checkpoint inhibitors (ICIs), has become a standard treatment for various malignancies and has demonstrated significant efficacy in some tumour types. However, the response of BTC to immunotherapy remains poor, with only approximately 5% of patients benefiting from single-agent immune checkpoint inhibitors (5). This low response rate is primarily due to the unique tumour microenvironment (TME) of BTC, which is highly immunosuppressive and characterized by the accumulation of tumour-associated macrophages (TAMs), regulatory T cells (Tregs), and myeloid-derived suppressor cells (MDSCs). These immunosuppressive cells play critical roles in tumour immune escape (6). As a result, single-agent immunotherapy has limited efficacy, necessitating the exploration of combination therapies to improve treatment outcomes.

Recent studies have proposed that reshaping the TME to reduce the number of immunosuppressive cells may significantly increase the efficacy of immunotherapy (7, 8). Compared with monotherapy, combination strategies that integrate immune checkpoint inhibitors with targeted therapies and chemotherapy have shown superior efficacy. Precision combination strategies, which combine molecular targeted therapy with immunotherapy, not only enhance immune responses but also overcome tumour resistance (9–11).

## 2 Molecular mechanisms and immune landscape of biliary tract cancer

### 2.1 Molecular heterogeneity in BTC

**Figure 1:**
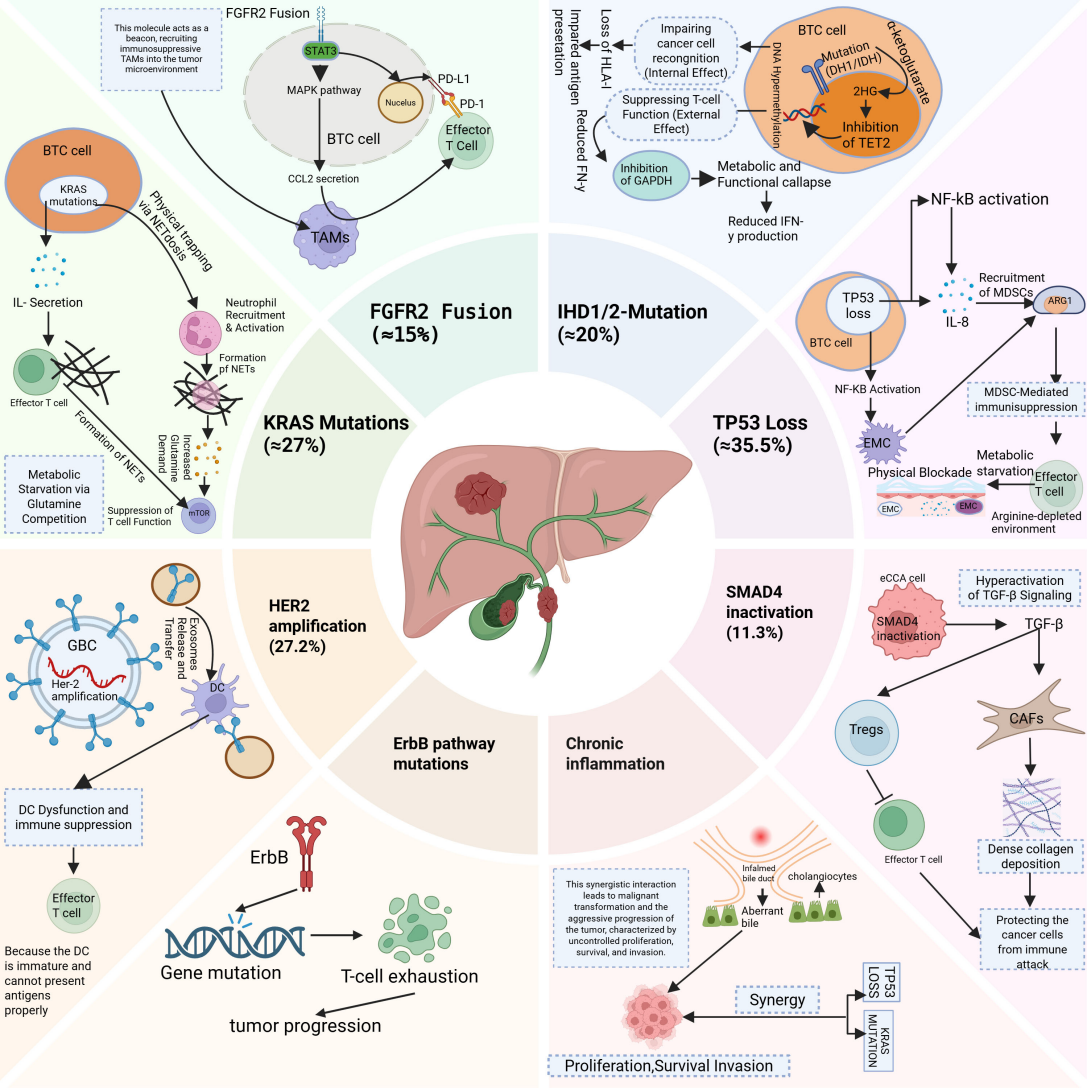
This diagram illustrates the genetic and molecular alterations in Biliary Tract Cancer (BTC) that contribute to immune evasion and tumour progression. BTC, Biliary tract cancer; TAMs, Tumour-associated macrophages; NETs, Neutrophil Extracellular Trap formation; MDSCs, Myeloid-derived suppressor cells; EMC, Extracellular matrix; GBC, Gallbladder cancer; DC, Dendritic cell; CAFs, Cancer-associated fibroblasts; Tregs, Regulatory T cells.

BTCs exhibit distinct genomic alterations that orchestrate oncogenic signalling and immune evasion. For example, FGFR2 fusions (≈15%) constitutively activate the MAPK/STAT3 pathways, upregulating PD-L1 via STAT3 binding to its promoter and recruiting immunosuppressive TAMs through CCL2 secretion, thereby establishing an immune-cold microenvironment (12). Similarly, IDH1/2 mutations (≈20%) drive the accumulation of (R)-2-hydroxyglutarate, which inhibits TET2-mediated demethylation and T-cell GAPDH activity, leading to impaired HLA-I antigen presentation and reduced IFN-γ production (13). In contrast, TP53 loss (35.5%) activates NF-κB-dependent IL-8 secretion, recruiting MDSCs that deplete arginine via ARG1 overexpression and deposit extracellular matrix (ECM) barriers to block T-cell infiltration (14–16). Moreover, KRAS mutations (≈27%) induce IL-8-mediated NETosis to physically trap T cells and compete for glutamine, suppressing mTOR-dependent T-cell function (17, 18). In GBC, HER2 amplification (27.2%) transfers HER2 to dendritic cells via tumour-derived exosomes, inhibiting DC maturation and antigen presentation (19), whereas SMAD4 inactivation in eCCA (11.3%) hyperactivates TGF-β signalling to directly promote Treg differentiation and collagen deposition by CAFs (20, 21). Notably, BRCA-deficient tumours (3.6%) presented elevated TMB (10.0 vs. 6.0 mut/Mb; *P*<0.001) and microsatellite instability (MSI-H: 17.9%), suggesting increased susceptibility to immune checkpoint blockade (22, 23). Overall, these driver mutations define molecular subtypes and offer therapeutic targets. Genomic heterogeneity stems from clonal evolution during chronic inflammation. In PSC-associated BTC specifically, TP53/KRAS mutations synergize with bile acid metabolic aberrations. This synergy drives malignancy (24, 25). Single-cell analyses revealed that ErbB pathway mutations in GBC promote tumour progression via T-cell exhaustion (26). Cellular origins further diversify ICC molecular profiles: Hepatocyte-derived iCCA frequently exhibits TERT promoter mutations, whereas cholangiocyte-derived tumours harbour BAP1 deletions (27–29). Epigenetic dysregulation (e.g., RBM10 splicing factor mutations) and homologous recombination defects (e.g., BRCA germline mutations) contribute to genomic instability (30, 31). In addition, spatial multiomics approaches, including single-cell sequencing and spatial transcriptomics, have revealed the significant intratumor heterogeneity present in BTCs. These technologies provide deeper insights into the complex cellular composition of tumours and their microenvironments, shedding light on how these heterogeneous regions influence treatment resistance (32, 33). For example, intratumor variation in immune cell infiltration and metabolic reprogramming has been linked to the development of resistance to immunotherapies and targeted therapies (33, 34). These findings underscore the importance of considering the spatial organization and functional diversity of tumours when therapeutic strategies are designed, as localized subpopulations within the tumour may exhibit differential responses to treatment (35, 36) (**Figure 1**).

### 2.2 Epigenetics in BTC

**Figure 2:**
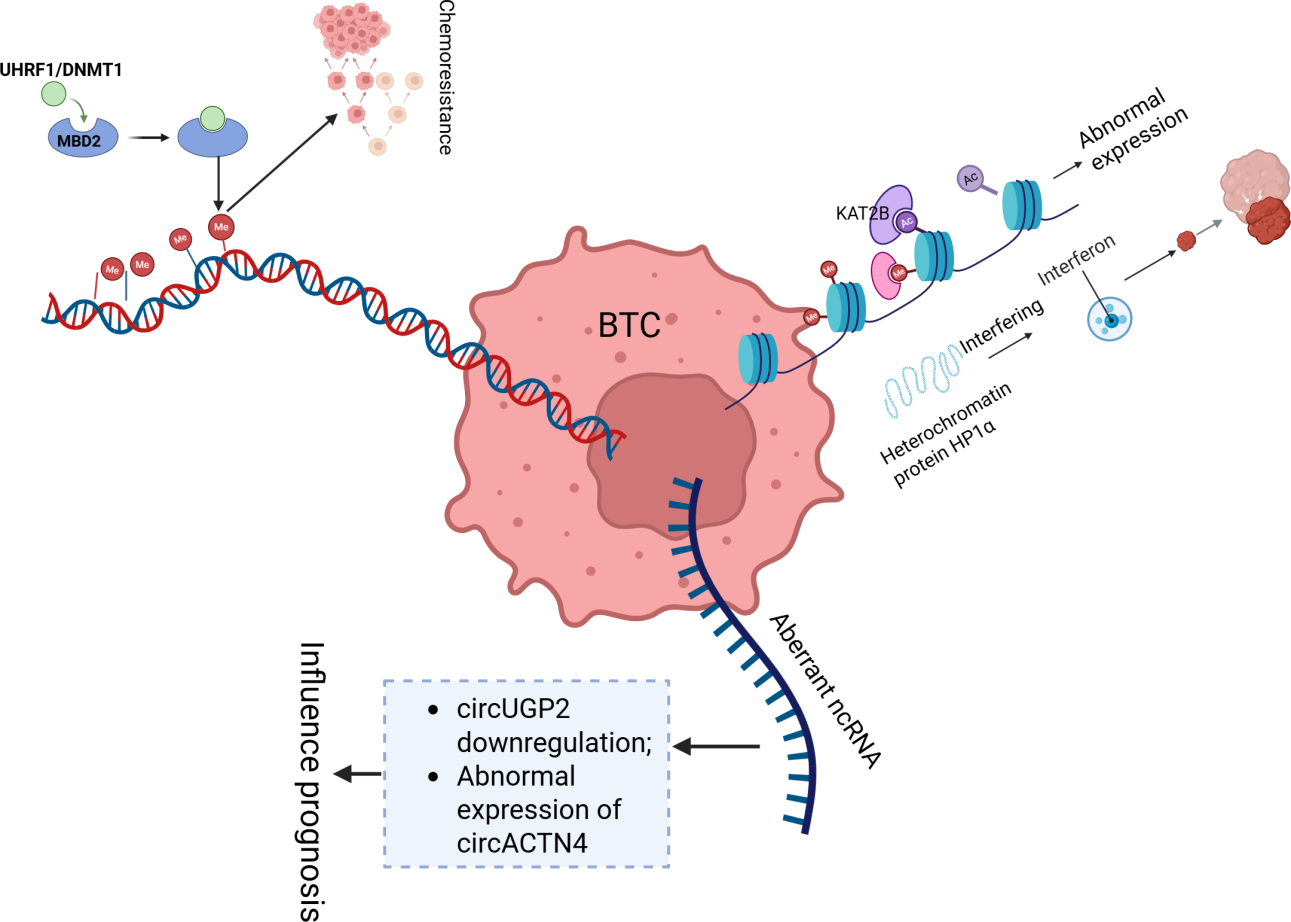
This diagram illustrates key epigenetic mechanisms involved in the development and progression of BTC. BTC, Biliary tract cancer; Me, Methylation; AC, Acetylation; ncRNA, Non-Coding RNA.

In the development and progression of BTC, abnormal DNA methylation, histone modifications, and noncoding RNA regulation form a complex epigenetic network. Abnormal DNA methylation, through the collaborative action of UHRF1/DNMT1 [by recruiting the HELLS chromatin remodelling complex to facilitate the recognition of hemimethylated CpG (37)] and methylation recognition mechanisms mediated by MBD2, drives chemotherapy resistance (38) and presents genome-wide differential methylation features across anatomical subtypes (iCCA, eCCA, and GBC) (39). In clinical practice, methylation markers in bile [such as early cholangiocarcinoma (CCA) markers in PSC patients (40)], differentially methylated regions (DMRs/DHMRs) in plasma circulating free DNA (cfDNA) (41, 42), and the F12 gene CpG site (43) can serve as liquid biopsy tools, improving diagnostic accuracy for malignant biliary strictures. Targeted therapies for IDH1 mutation-associated methylation abnormalities (44) and DNMT3A overexpression [persistently present in the progression from cholelithiasis to gallbladder cancer (45, 46)] have shown potential for personalized treatment (47, 48). With respect to histone modifications, abnormal expression of the histone acetyltransferase KAT2B (49) and methyltransferase G9a [which promotes BTC invasion through the Hippo pathway LATS2/YAP regulation (50, 51)] drives tumour progression, whereas the heterochromatin protein HP1α regulates iCCA proliferation by interfering with the interferon pathway (52). Among noncoding RNAs, downregulation of circUGP2 (53) and abnormal expression of circACTN4 and cPKM [which promote chemotherapy resistance through the PKM2/β-catenin axis (38)] influence prognosis. LINC00511 (54) and miR-27a-3p [targeting the FOXO1/PI3K/AKT pathway (55, 56)] regulate tumour stem cell properties, whereas exosomal circRNAs [such as bile-derived CCA-circ1 (57)] and circNFIB [through miR-412-3p/PIK3R3 inhibition of metastasis (58)] can serve as molecular subtype biomarkers. These mechanisms, which are mediated by RNA splicing via RBM10 (59) and signalling pathways such as the PI3K/AKT pathway (60), lay the foundation for prognosis evaluation and targeted therapy development in BTC (**Figure 2**).

### 2.3 Changes in the tumour microenvironment

In iCCA, TAMs play a pivotal role in driving immunosuppression by upregulating PD-L1, which promotes T-cell exhaustion (61). This process is further enhanced by abnormally activated regulatory T cells (Tregs), which decrease antitumour immunity by suppressing effector T-cell function and the secretion of proinflammatory cytokines, such as IL-10 and TGF-β (62). The cross-talk between TAMs and Tregs creates a mutually reinforcing immunosuppressive loop in which TAMs secrete factors such as IL-6 and GM-CSF, which can promote Treg expansion and activation (63–65). IL-6, produced by both tumour and stromal cells, not only enhances cancer stem cell (CSC) proliferation but is also a key mediator of systemic inflammation, which is correlated with poor prognosis in CCA patients (66, 67). IDH1-mutant tumours exacerbate immune suppression through metabolic reprogramming, increasing the production of metabolites such as 2-HG, which inhibits the function of TET2 and reduces T-cell activation, contributing to resistance against ICIs (68–70). Furthermore, VEGF-C-driven lymphangiogenesis contributes to an immunosuppressive microenvironment by facilitating lymph node metastasis and further suppressing immune responses through the recruitment of immunosuppressive cells (71, 72).

**Figure 3:**
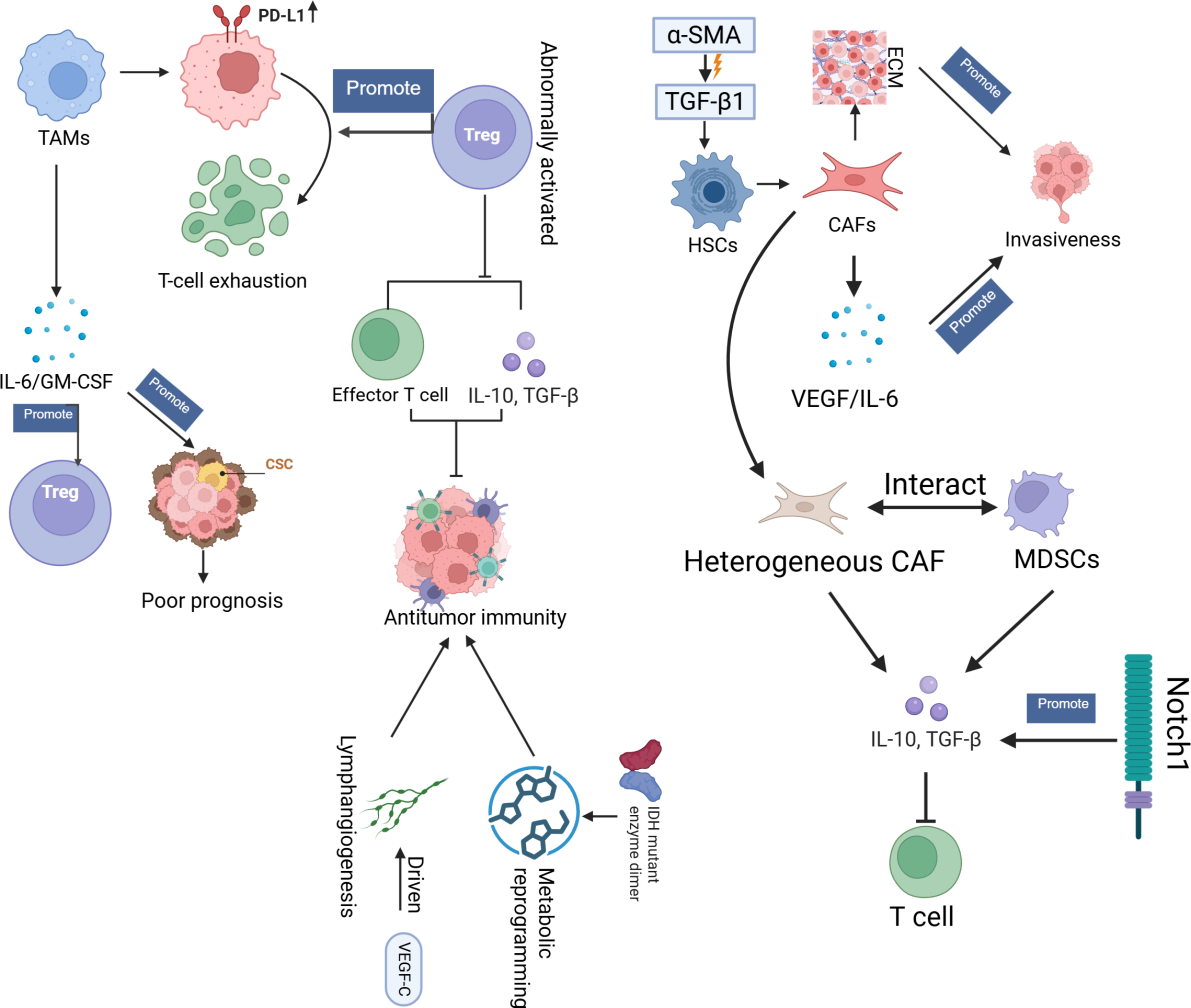
This diagram provides an overview of the TME in BTC, highlighting key cellular and molecular interactions that drive immune evasion and tumour progression. TME, tumour microenvironment; CAFs, Cancer-Associated Fibroblasts; TAMs, Tumour-Associated Macrophages, Tregs, Regulatory T-cells; MDSCs, Myeloid-Derived Suppressor Cells; EMC, Extracellular matrix; HSCs, Hepatic Stellate Cells; CSC, Cancer stem cells.

Additionally, cancer-associated fibroblasts (CAFs), derived from TGF-β1-activated hepatic stellate cells (HSCs) that express α-SMA, play a critical role in remodelling the TME. CAFs secrete extracellular matrix (ECM) components that create mechanical barriers to promote invasiveness and chemoresistance in iCCA (73–75). CAFs not only increase tumour progression through the secretion of protumour factors, such as VEGF and IL-6, but also induce vasculogenic mimicry (VM), which accelerates metastasis in gallbladder cancer (67, 76, 77). Heterogeneous CAF subpopulations interact with myeloid-derived suppressor cells (MDSCs) and inhibit T-cell function by secreting immunosuppressive cytokines, such as IL-10 and TGF-β. This interaction is facilitated by the Notch1 signalling pathway, which not only amplifies tumour malignancy but also helps CAFs recruit MDSCs to the TME (78, 79). CAFs can transfer oncogenic molecules, such as miRNAs and proteins, via exosomes, further contributing to resistance in genomically distinct subtypes, including ERBB2-amplified tumours (80–82). These interactions between CAFs, TAMs, Tregs, and MDSCs create a complex immune-suppressive network that supports immune evasion and tumour progression in cholangiocarcinoma. These findings underscore the importance of targeting the functional diversity of these immune cells using combinatorial strategies, such as Notch inhibitors or immunotherapies, to overcome resistance and improve therapeutic outcomes (65, 83) (**Figure 3**).

### 2.4 Tumour-induced immune evasion mechanisms

**Figure 4:**
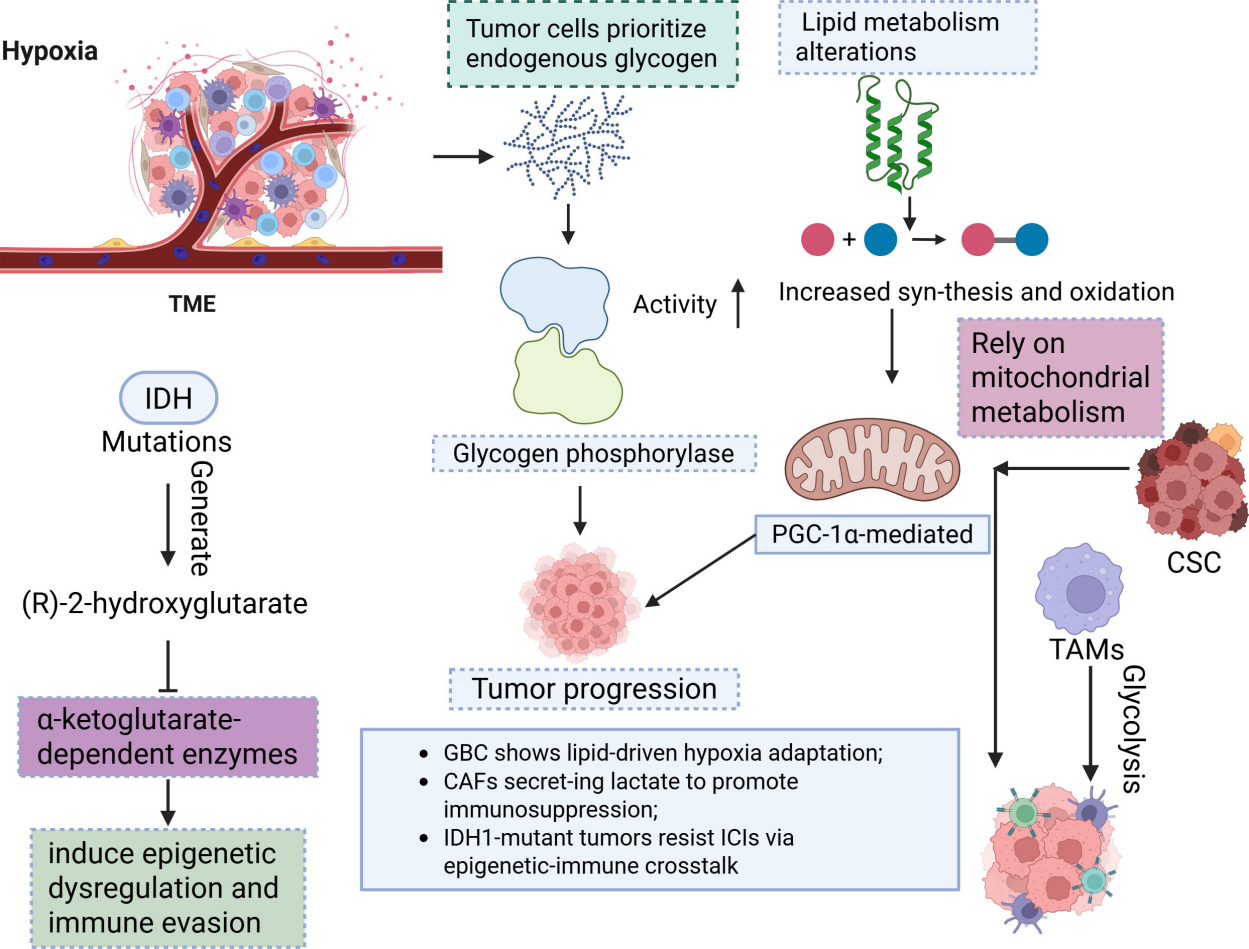
This diagram depicts the metabolic reprogramming in BTC driven by hypoxia and IDH mutations. TME, Tumour microenvironment; CSC, Cancer stem cells; TAM, Tumour-Associated Macrophages.

Multiomics remodelling has revealed profound metabolic heterogeneity in ICCA, including enhanced glycolysis, lipid dysregulation, and mitochondrial adaptation (84–86). Tumour cells prioritize endogenous glycogen over glucose as the primary glycolytic carbon source under hypoxia, with elevated glycogen phosphorylase activity fuelling tumour progression (84). Lipid metabolism alterations, such as increased synthesis and oxidation, and PGC-1α-mediated mitochondrial reprogramming sustain proliferation and represent therapeutic vulnerabilities (87). IDH1 mutations generate (R)-2-hydroxyglutarate, which inhibits α-ketoglutarate-dependent enzymes to induce epigenetic dysregulation and immune evasion (68). Cancer stem cells (CSCs) rely on mitochondrial metabolism, whereas TAMs exacerbate immunosuppression via glycolytic shifts (86, 87). These metabolic adaptations correlate with anatomical subtypes (e.g., ERBB2-amplified iCCA vs. PIK3CA-mutant extrahepatic tumours) and influence chemotherapy/targeted therapy efficacy (88–90). Multiomics analyses further revealed metabolic heterogeneity across subtypes: gallbladder cancer shows lipid-driven hypoxia adaptation, whereas extrahepatic tumours exhibit PI3KCAH1047R-driven transformation (90–92). Metabolic crosstalk within the TME involves CAFs that secrete lactate to promote immunosuppression and TAMs that enhance glycolysis to suppress immunity (53, 93, 94). Exosome-mediated signalling coordinates angiogenesis and fibrosis, whereas metabolic–immune network dysregulation (e.g., GPR109A pathway anomalies) underpins poor prognosis and therapy resistance (95–97). IDH1-mutant tumours resist ICIs via epigenetic–immune crosstalk, whereas CPS1-deficient tumours disrupt the urea cycle to alter the pH of the TME (98–100). Targeting metabolic nodes, such as glycolytic enzymes, mitochondrial pathways, or FGFR2/IDH1-related aberrations, may reverse immunosuppression and increase treatment sensitivity (18, 53, 94, 101). Overall, the bidirectional feedback between metabolic reprogramming and TME remodelling drives cholangiocarcinoma progression, necessitating precision strategies that integrate metabolic subtyping with immune and targeted therapies (86, 102, 103) (**Figure 4**).

## 3 Current immunotherapy approaches for BTC

### 3.1 Monotherapy with immune checkpoint inhibitors

ICIs, particularly PD-1/PD-L1 inhibitors and CTLA-4 inhibitors, are among the most successful immunotherapy strategies. PD-1/PD-L1 inhibitors work by disrupting immune suppression between tumour cells and immune cells, promoting the activation of T cells and enhancing antitumour immune responses (104–106). PD-1 inhibitors, such as nivolumab and pembrolizumab, have entered clinical trials for the treatment of BTC and have shown some efficacy (107, 108). This finding indicates the limitations of monotherapy in BTC and highlights the immune suppressive characteristics of the tumour microenvironment in this cancer.

### 3.2 Combination therapies

The combination of immune checkpoint inhibitors with chemotherapy has been increasingly investigated in BTC, with preliminary results suggesting that this combination can significantly improve clinical outcomes. For example, the combination of pembrolizumab with gemcitabine/cisplatin has improved progression-free survival (PFS) and overall survival (OS) in advanced BTC patients compared with chemotherapy alone (3). This combination approach harnesses the cytotoxic effects of chemotherapy while activating the immune response through immune checkpoint inhibitors, providing a more effective treatment option for BTC patients (109). Combining immunotherapy with targeted therapy is another promising strategy currently being explored for BTC. The combination of PD-1 inhibitors with FGFR2 inhibitors or IDH1 inhibitors aims to enhance immune responses and overcome tumour resistance mechanisms. For example, the combination of PD-1 inhibitors with FGFR2 inhibitors has shown potential in clinical trials, with some patients experiencing delayed disease progression (88, 110–113). These combination therapies aim to target both immune escape pathways and molecular drivers of tumour growth, offering a multipronged approach to improve BTC treatment outcomes.

These combination strategies offer several advantages in BTC treatment. First, they increase treatment efficacy by simultaneously targeting immune evasion pathways and molecular drivers of tumour growth, which improves clinical outcomes. Mechanistic studies support these clinical findings: Mechanistically, targeting TAMs and their PD-L1 upregulation can effectively alleviate immune suppression and promote T-cell-mediated tumour killing (114). Additionally, combining chemotherapy with immune checkpoint inhibitors synergistically amplifies the therapeutic response and improves PFS and OS (115). In addition, the combination of targeted therapies with immunotherapy helps overcome tumour resistance mechanisms, providing a more comprehensive treatment approach. Targeted drugs, such as FGFR2 inhibitors and IDH1 inhibitors, block tumour cell proliferation and signalling pathways, thus increasing the effectiveness of immunotherapy and restoring the ability of the immune system to recognize tumours (116, 117). This multifaceted strategy aims to address the complexity of BTC and may increase the sustainability of clinical responses.

However, combination therapy also faces several challenges. The combined use of chemotherapy and immune checkpoint inhibitors may increase toxicity and immune-related adverse events (irAEs), which may limit the tolerability of these treatments in some patient populations (115). Additionally, tumour heterogeneity in BTC is a significant challenge because the molecular characteristics of a patient may dictate the response to combination treatment. Mechanistic research suggests that molecular features, such as FGFR2 and IDH1 mutations, may influence immune evasion and resistance mechanisms (118, 119). Further research on the relationship between tumour molecular characteristics and treatment response is crucial to optimize combination therapies. Finally, reliable biomarkers to identify patients who will benefit the most from combination therapies are lacking, and the discovery of such biomarkers remains a significant challenge. Mechanistic studies have pointed to the potential of identifying biomarkers, such as key molecules in the tumour microenvironment, which may help optimize patient selection. Ongoing research is needed to identify suitable biomarkers for patient selection and refine these combination treatment strategies. Despite these challenges, combination therapy remains a promising avenue for improving BTC treatment outcomes (120), and further clinical trials and mechanistic studies are necessary to optimize these approaches.

## 4 Clinical trials and applications in BTC

Currently, clinical trials on immunotherapy in BTC are ongoing, and many are evaluating the effects of combining immunotherapy with chemotherapy and targeted therapies. As research advances, more immunotherapy regimens are expected to enter clinical practice, particularly in the areas of precision medicine and individualized treatment. Moreover, new immunotherapy targets and strategies that combine immunotherapy with metabolic modulation are being explored, and future research is expected to identify more treatment options for BTC patients (**Table 1**).

**Table 1:** Clinical trials of BTC.

Trial ID	Phase (Estimated number); line	Tumour type	Molecular target	Treatment type	Status	Last update posted	Primary outcome	Location	Results
NCT04466891	IIb(N=87); 1st	GBC	Her2	Zanidatamab	Completed	Aug 21, 2024	ORR	US	ORR:41.3%
NCT01308840	II(N=31);1st	BTC; GBC	Kras/B-raf mutations	GEMOX-Panitumumab	Completed	Aug 21, 2024	ORR	US	ORR:50%; mPFS:10.5 months; mOS:24.8 months ORR:41.3%
NCT02265341	II(N=12);1st	BTC	FGFR2 fusion	Ponatinib Hydrochloride	Completed	Aug 6, 2019	CBR (≥4 moth)	US	CBR:5%
									ORR:20.7%;
									DCR82.8%
NCT01752920	I-II(N=29)1st	iCCA	FGFR genetic aberrations; FGFR2 gene fusion	Derazantinib	Completed	June 5, 2023	ORR; DCR	US	
NCT03230318	II(N=29)2nd	iCCA	FGFR2 fusions; FGFR2 mutations	Derazantinib	Completed	Dec 19, 2023	ORR; DCR; mPFS	US	ORR:20.7%; DCR:82.8%; mPFS:5.7 months
NCT02924376	II(N=)2nd	BTC	FGFR2 translocation	Pemigatinib	Completed	ORR; DCR; mDOR	Feb 23, 2023	US	ORR:37.0%; DCR:82.4%; mDOR9.1 months
NCT02034110	II(N=43)1st	BTC	BRAF V600E mutation	Dabrafenib+ Trametinib	Completed	Aug 21, 2023	Best Response	US	CR:2%; PR:53%; SD:35%
NCT01935843	I(N=11)1st	PCs; BTC	HER2-positive	CART-HER2 cell	Completed	Jan 28, 2016	PFS	China	PFS:4.5months
				nab-paclitaxel					
				Cyc					
NCT04088188	I (N=8)1s	BTC	IDH	Pemigatinib; Cisplatin; Gemcitabine; Ivosidenib	Completed	May 6, 2024	OS;PFS	US	mOS(22.9 months vsNA);mPFS (15.4months vs 4.9months)
NCT04722133	I(N=34)2nd	BTC	HER2-positive	Trastuzumab	Completed	Oct 11, 2023	ORR; DCR	Korea	ORR:29.4%; DCR:79.4%
				FOLFOX					
NCT02989857	III(N=185)3rd	BTC	IDH1 mutations	Ivosidenib	Completed	Aug 20, 2024	mPFS;	US	mPFS(2.7 months vs1.4months)
NCT04329429	II(N=57)2nd	BTC	HER2 Overexpressed	RC48-ADC	Active, not recruiting	December 20, 2023	Best Response	China	Results pending
NCT04579380	II(N=217)1st	Solid Tumours; BTC	HER2 alterations	Tucatinib; Trastuzumab; Fulvestrant	Active, not recruiting	March 25, 2025	ORR	China	Results pending
NCT06282575	III (N=286)	BTC	HER2	Zanidatamab+ CisGem	Recruiting	May 13, 2025	OS	US	Results pending

GEMOX, Gemcitabine+ Oxaliplatin; N, None; ADC, Antibody-Drug Conjugate; Cyc, cyclophosphamide; PC, Pancreatic cancers; iCCA, Intrahepatic Cholangiocarcinoma; GBC, Gallbladder Cancer; BTC, Biliary Tract Cancer; ORR, Overall Response Rate; PFS, Progression-Free Survival; OS, Overall Survival; DCR, Disease Control Rate; CR, Complete Response; SD, Stable Disease.

## 5 Reshaping the TME

### 5.1 TME Components and their impact on immunotherapy

Critically, TAMs are the predominant immune cells in the BTC TME. They play pivotal roles in promoting both tumour progression and immune suppression. TAMs secrete cytokines, such as IL-6 and TNF-α, that inhibit T-cell activation while promoting the proliferation of cancer stem cells. Moreover, TAMs upregulate immune checkpoints, such as PD-L1, which further suppresses CD8+ T-cell activity, thereby facilitating immune escape and promoting tumour growth (87, 121, 122). Tregs are another crucial cell type in the BTC TME that significantly contribute to immune suppression. Tregs secrete immunosuppressive cytokines, such as TGF-β and IL-10, to directly inhibit the function of effector T cells (e.g., CD8+ T cells). This action suppresses antitumour immunity. The accumulation of Tregs in the BTC TME is associated with poorer prognosis, indicating their role in immune evasion and resistance to treatment (123–125). MDSCs constitute another class of immune-suppressive cells that accumulate in the TME under the influence of tumour-secreted factors. MDSCs suppress T-cell activation and promote tumour progression by secreting immunosuppressive molecules, such as arginase-1 (Arg1) and inducible nitric oxide synthase (iNOS) (126). In BTC, the presence of MDSCs is correlated with immune suppression and disease progression (121, 127). CAFs are a key cell type in the TME and contribute to tumour progression through the secretion of ECM components (such as collagen and fibronectin) and protumourigenic factors (such as VEGF and IL-6). CAFs remodel the ECM, which can create physical barriers that impede immune cell infiltration. In addition, CAFs promote immune evasion by secreting lactate and other metabolic products, further suppressing immune responses (128).

### 5.2 Strategies to reshape the TME

One approach to reshaping the TME involves targeting immune-suppressive cells, such as TAMs, Tregs, and MDSCs. TAMs can be targeted using antibodies against CD40 or CSF1R, which reduce the number of TAMs and restore immune responses (129, 130). Targeting Tregs using anti-CTLA-4 antibodies or anti-CCR4 antibodies, which can deplete Tregs and promote effector T-cell function, has been proposed as another strategy to reshape the TME (131). Additionally, targeting MDSCs with anti-GM-CSF antibodies can reduce their accumulation and reverse immune suppression in the TME (130). The dense ECM in the TME often acts as a physical barrier that prevents immune cells from infiltrating tumour tissues. Strategies to target ECM components are being developed to increase immune cell infiltration. For example, the use of matrix metalloproteinase (MMP) inhibitors has been shown to degrade the ECM barrier, promoting immune cell entry and enhancing the effects of immunotherapy (132). Furthermore, targeting ECM components secreted by CAFs can help alleviate mechanical barriers in the TME and improve immune responses (133, 134). The metabolic reprogramming of both tumour and immune cells in the TME plays a crucial role in immune evasion. The acidic and hypoxic conditions in the TME, which are caused by altered metabolic pathways, suppress immune cell function. Targeting these metabolic pathways in tumour and immune cells, such as glycolysis and lipid metabolism, has been proposed as a strategy to restore immune function and increase the efficacy of immunotherapy (135, 136). For example, inhibiting lactate metabolism in tumour cells can reverse immune suppression and enhance the antitumour effects of immune cells (137).

### 5.3 Combination of immunotherapy and TME reshaping

Combining immunotherapy with strategies to reshape the TME has shown promising results in preclinical models. Targeting both immune-suppressive pathways in the TME and enhancing immune responses has synergistic effects (138). For example, combining PD-1 inhibitors with CAF-targeting therapies has resulted in increased antitumour effects in preclinical studies (139). This approach not only improves immune cell function but also suppresses tumour growth, providing a more effective treatment strategy for BTC.

## 6 Advancing precision combination strategies

### 6.1 Combination of immunotherapy with targeted therapy

FGFR2 gene fusions or amplifications are common in iCCA, with approximately 10–15% of iCCA patients harbouring FGFR2 alterations. FGFR2 inhibitors, such as pemigatinib and futibatinib, have shown significant efficacy in FGFR2 fusion-positive patients (93, 140). Combining FGFR2 inhibitors with PD-1 inhibitors (e.g., nivolumab) may enhance the effects of immunotherapy. This potential benefit arises because FGFR2 inhibitors inhibit tumour cell proliferation. Moreover, they enhance immune system responses (141). IDH1 mutations occur in a subset of BTC patients, particularly in iCCA patients. IDH1 inhibitors (e.g., ivosidenib) restore normal metabolic pathways, inhibiting tumour cell growth. In early clinical trials, the combination of IDH1 inhibitors with immunotherapy has shown potential for enhancing immune responses (142). HER2 overexpression or amplification is observed in some gallbladder cancers and iCCA. HER2-targeted therapies (e.g., trastuzumab) in combination with immunotherapy (e.g., PD-1 inhibitors) may enhance antitumour immune responses and overcome immune evasion (55, 143).

### 6.2 Combination of immunotherapy with chemotherapy

The combination of immune checkpoint inhibitors, such as nivolumab or pembrolizumab, with chemotherapy (e.g., gemcitabine and cisplatin) has shown superior efficacy in BTC treatment (3). Compared with chemotherapy alone, the combination of PD-1 inhibitors with chemotherapy significantly improves PFS and OS. Additionally, immunotherapy helps overcome chemotherapy resistance, enhancing immune responses against tumours (144). Chemotherapy induces tumour cell death, leading to the exposure of tumour antigens, which enhances immune system recognition of the tumour (145). Moreover, chemotherapy increases immune cell infiltration into the tumour, creating a more favourable environment for immunotherapy. The combination of chemotherapy and immune checkpoint inhibitors not only enhances immune responses but also provides complementary mechanisms to combat tumour growth, improving therapeutic efficacy (105, 107).

### 6.3 Combination of immunotherapy with metabolic modulation

Tumour cells enhance glycolysis and lipid metabolism to promote growth while suppressing immune cell function. Inhibiting tumour cell glycolysis or lipid biosynthesis restores immune cell antitumour functions, increasing the efficacy of immunotherapy. The combination of metabolic inhibitors with immunotherapy has demonstrated significant synergistic effects in preclinical models, particularly in BTC models (3, 146). Metabolic reprogramming in the TME creates an acidic and hypoxic environment that suppresses immune cell activity. Targeting these metabolic pathways and restoring normal metabolic states may reverse the immune-suppressive environment in the TME, thereby enhancing the effects of immunotherapy (147). For example, the use of glucose metabolism inhibitors or lactate removal agents has been shown to effectively restore antitumour responses of the immune system and improve immunotherapy response rates (148).

## 7 Challenges in clinical translation

Despite advancements in targeted and immunotherapies, several challenges hinder their clinical translation for BTC. The molecular heterogeneity of BTC, with varying genetic profiles across subtypes (iCCA, eCCA, and GBC), complicates the identification of universal therapeutic targets. The difficulty in obtaining sufficient tissue samples for molecular profiling is addressed by liquid biopsy technologies, particularly cfDNA, which offer a noninvasive method to monitor mutations and treatment responses in real time (149). The immunosuppressive tumour microenvironment, dominated by Tregs, MDSCs, and TAMs, limits the efficacy of ICIs. Overcoming this barrier requires strategies such as combining ICIs with immunomodulatory agents. Additionally, the development of predictive biomarkers, such as tumour mutational burden and specific genetic alterations, is essential for patient selection and optimizing treatment strategies.

Chemotherapy, targeted therapy, and immunotherapy each offer distinct advantages and limitations in the management of advanced CCA (150). Chemotherapy remains a cornerstone of treatment, particularly for advanced cases, but its effectiveness is often limited by systemic toxicity and a lack of durable responses (151). Targeted therapies, such as those targeting specific genetic alterations such as FGFR2 fusions or IDH mutations, have shown promising results in selected patients but are limited by the genetic heterogeneity of CCA and the development of resistance over time (152). Immunotherapy, particularly ICIs, offers a new approach by targeting immune evasion mechanisms within the tumour microenvironment (153). However, the immunosuppressive nature of the CCA microenvironment, which is dominated by regulatory T cells (Tregs), myeloid-derived suppressor cells (MDSCs), and TAMs, limits the effectiveness of ICIs in many patients (154). Overcoming this challenge may require combining ICIs with immunomodulatory agents or other treatment strategies to enhance the immune response (155). Furthermore, predictive biomarkers, such as tumour mutational burden (TMB) and specific genetic alterations, are crucial for patient selection and optimizing the therapeutic approach, ensuring that patients most likely to benefit from each modality receive the appropriate treatment (156).

## 8 Overcoming clinical challenges in immunotherapy for BTC

### 8.1 Mechanisms of immunotherapy resistance

BTC tumour cells evade immune surveillance through several mechanisms. These processes primarily involve 1) the upregulation of immune checkpoints (such as PD-L1 and CTLA-4), 2) the accumulation of TAMs and regulatory Tregs, and 3) the reprogramming of tumour metabolic pathways. These mechanisms limit the immune response and contribute to tumour immune escape, thereby exacerbating resistance (157). For example, high PD-L1 expression in BTCs directly inhibits CD8+ T-cell-mediated antitumour effects (158). TAMs and Tregs, through the secretion of immunosuppressive cytokines such as IL-10 and TGF-β, further weaken immune responses (159). Metabolic reprogramming in the TME is another important factor leading to immune evasion. BTC cells promote glycolysis, lipid synthesis, and lactate secretion, creating an acidic, hypoxic, and nutrient-deprived microenvironment. This environment not only provides a growth advantage to tumour cells but also suppresses immune cell functions. For example, the accumulation of lactate in the TME impairs T-cell efficacy and suppresses immune responses (160).

### 8.2 Immune-related adverse events

Although ICIs have demonstrated efficacy in various cancers, immune-related adverse events (irAEs) associated with these treatments have become a significant clinical challenge. irAEs refer to autoimmune reactions triggered by the activation of the immune system, which can result in damage to various organs, including the skin, liver, lungs, and endocrine system (161, 162). In BTC patients, the incidence of irAEs due to ICIs is relatively high, and their management requires careful consideration of the patient’s immune status and comorbidities. This management is especially challenging in patients with advanced BTC or those with multiorgan metastasis, where irAEs complicate treatment. Therefore, balancing efficacy with the management of irAEs remains a major clinical challenge in BTC immunotherapy.

The management of irAEs in BTC patients involves a range of strategies aimed at controlling immune overactivation while maintaining the antitumour efficacy of ICIs. First-line treatment typically involves systemic corticosteroids, with additional immunosuppressive agents such as mycophenolate mofetil or infliximab for severe cases (163–165). Temporary interruption of ICIs may be recommended for mild irAEs, with reintroduction upon symptom resolution. Preventive measures focus on pretreatment screening to identify high-risk patients, particularly those with a history of autoimmune disorders. Low-dose corticosteroids before treatment and personalized monitoring using biomarkers predictive of immune activation are areas of active research. Although these strategies can reduce the severity of irAEs, further clinical validation is needed before they can be widely implemented.

Balancing efficacy with safety is a critical challenge in BTC immunotherapy, especially given the potential for severe irAEs. To optimize outcomes, clinicians must tightly monitor patients by assessing organ function and tracking symptoms regularly. An individualized approach is necessary, wherein the decision to continue or pause ICI therapy is based on the patient’s overall health, the severity of irAEs, and potential survival benefits (166). Furthermore, combining ICIs with other therapies, such as chemotherapy or targeted treatments, may help mitigate the immune activation seen with monotherapy, potentially reducing the risk of irAEs while retaining treatment efficacy (167, 168). Ongoing research into biomarkers to predict irAEs and methods to prevent their occurrence will be essential for improving the safety profile of ICIs in BTC and enhancing the clinical management of these patients (169).

### 8.3 Patient selection and the lack of biomarkers

Currently, the response rate of BTC patients to immunotherapy remains low, making patient selection crucial. However, accurately identifying patients who are most likely to benefit from immunotherapy is difficult due to the lack of effective predictive biomarkers. While some biomarkers, such as PD-L1 expression, microsatellite instability (MSI), and the TMB, have been shown to have predictive value in other cancers, their utility in BTC remains unvalidated (170, 171). Biomarker identification for immunotherapy response in BTC remains in its early stages. Emerging technologies, such as liquid biopsy, ctDNA, and genomic analysis, offer the potential for earlier and more accurate assessment of treatment efficacy and resistance. Furthermore, research suggests that the interaction between immunotherapy and the TME may affect the treatment response, highlighting the potential of TME components as predictive biomarkers (172).

### 8.4 Challenges in TME reshaping

Although reshaping the TME to increase immunotherapy efficacy has become a significant research focus, translating this strategy into clinical practice still faces multiple challenges. First, the complexity and heterogeneity of the TME hinder the complete elimination of immunosuppressive components (172, 173). Additionally, TME-reshaping drugs may interact with immunotherapies, potentially causing side effects. For example, targeting CAFs (cancer-associated fibroblasts) may lead to adaptive changes in tumour cells that result in therapy resistance (174). Therefore, precisely targeting immune-suppressive components within the TME while minimizing side effects and maximizing the effectiveness of immunotherapy remains a key focus for future research.

### 8.5 Limitations and challenges in clinical trials

Although numerous clinical trials related to immunotherapy are underway, some limitations still remain in the design of clinical trials for BTC. First, most clinical trials have focused on late-stage BTC patients, often overlooking patients with early-stage or locally advanced disease. Second, the heterogeneity of trial results is significant, due in part to individual patient differences, tumour heterogeneity, and variations in immune responses. Therefore, larger, more rigorously designed clinical trials are needed to validate the true effects and safety of immunotherapy in BTC (175) **Table 2**.

**Table 2:** Clinical trials of immunotherapy for biliary tract cancer.

Trial ID	Phase (Estimated number); line	tumour Type	Molecular target	Treatment type	Status	Last update posted	Primary outcome	Location	Results
NCT03101566	II(N=75);1st	BTC	/	Cisplatin	Completed	Feb 23, 2023	PFS;	US	PFS: A 59.4% B 21.2% OS:A:6.6months VS 10.6months
				Gemcitabine			OS		
				Nivolumab					
				Ipilimumab					
									OS:B 3.9months VS 8.2months
							
							
							
NCT04677504	II(N=162)1st	BTC	/	Atezolizumab	Completed	July 3, 2024	mPFS	US	PFS: 8.4months (atezo+bev+CisGem) VS 7.9months (atezo+ placebo+CisGem)
				Bevacizumab					
					
							
							
NCT02703714	II(N=21)2nd	BTC	/	MK3475 + GM-CSF	Completed	Jan 25, 2022	ORR	US	ORR:21%
					
NCT03875235	III (N= 341 vs 344) 1st	BTC	/	Durvalumab + GP chemotherapy VS placebo + GP Chemotherapy	Completed	Mar 25, 2025	ORR	US	ORR: 22.9% vs 3%
				
							
							
NCT03796429	II(N=50)1st	BTC	/	Toripalimab	Completed	Nov 29, 2023	ORR	China	ORR:30.6%
				Gemcitabine					
				S1					
NCT03486678	II(N=38)1st	BTC	/	Camrelizumab	Completed	Oct 28, 2021	PFS, OS	China	PFS:6.1months, OS:11.8months
				GEMOX					
NCT02443324	I(N=26)2nd	BTC	/	Pembrolizumab		July 31, 2024	ORR; PFS; OS	UK	ORR:4%; PFS:6.1months; OS:6.4months
				Ramucirumab					
						
NCT03892577	Real-world Study(N=31)1st	GBC	/	PD-1 inhibitors	Completed	Mar 29, 2023	ORR	China	ORR:32.3%
				Lenvatinib					
NCT03951597	II(N=30)1st	BTC	/	Toripalimab	Completed	July 7, 2021			
				lenvatinib			ORR	China	ORR:80%
				GEMOX					
NCT01853618	II(N=30)2nd	BTC	/	Tremelimumab	Completed	Dec 10, 2019	ORR	US	ORR:12.5%
				microwave ablation					
							
NCT03482102	I(N=30)2nd	BTC	/	Durvalumab/	Completed	Sep 08, 2022	ORR	US	ORR:20%
				Tremelimumab					
				Radiotherapy					
NCT03110328	I(N=33)2nd	Meta BTC	/	MK3475	Completed	Jun 12, 2022	ORR	Korea	Results pending

Met, Metastatic; MK3475, pembrolizumab; GEMOX, Gemcitabine+ Oxaliplatin; GBC, Gallbladder Cancer; BTC, Biliary Tract Cancer; ORR, Overall Response Rate; PFS, Progression-Free Survival; OS, Overall Survival; DCR, Disease Control Rate; CR, Complete Response; SD, Stable Disease.

## 9 Future directions and emerging therapies

### 9.1 Next-generation immunotherapies

Chimeric antigen receptor T-cell (CAR-T) therapy is an emerging treatment strategy that involves genetically modifying a patient’s own T cells to recognize and attack tumour cells. Although CAR-T-cell therapy has achieved significant success in haematologic cancers, its application in solid tumours, including BTCs, faces challenges. Research indicates that the primary challenge for CAR-T-cell therapy in solid tumours is the ability to effectively penetrate the tumour microenvironment and enhance T-cell infiltration (176, 177). Current research focuses on optimizing CAR-T-cell therapy design, including targeting specific antigens found in BTCs, such as Mucin 1 and CEA, to improve efficacy and reduce side effects (178). Tumour vaccines, particularly mRNA-based vaccines, constitute another emerging strategy in immunotherapy. mRNA vaccines deliver specific tumour antigen genes to the patient’s body, inducing the immune system to recognize and attack tumour cells. mRNA vaccines that target BTCs are currently in the preclinical phase, and these vaccines have the potential to enhance antitumour immune responses (179). For example, mRNA vaccines that target KRAS mutations have shown therapeutic potential in other solid tumours and may be used for BTC treatment in the future (180).

### 9.2 Targeting novel immune checkpoints

T-cell immunoreceptor with Ig and ITIM domains (TIGIT) and lymphocyte-activation gene 3 (LAG-3) are newly identified immune checkpoint molecules that play critical roles in immune suppression in various cancers. Research indicates that the upregulation of TIGIT and LAG-3 is closely associated with immune evasion and tumour resistance (181–183). Currently, monoclonal antibodies that target TIGIT and LAG-3 are undergoing clinical trials. Combining PD-1/PD-L1 inhibitors with TIGIT or LAG-3 inhibitors may improve treatment responses in BTC patients (184). V-domain Ig suppressor of T-cell activation (VISTA) is another newly discovered immune checkpoint molecule that plays a key role in regulating T-cell function (185). VISTA upregulation in the TME has been shown to play a role in immune suppression, particularly in solid tumours. Antibodies that target VISTA are currently under development and may offer new treatment options for BTC patients when combined with existing immunotherapies (186).

### 9.3 Artificial intelligence and immunotherapy

AI and deep learning technologies can analyse patient genomic data, immune phenotypes, and clinical characteristics to identify biomarkers associated with immunotherapy response. These technologies can help clinicians select the patients most likely to benefit from immunotherapy and provide personalized treatment plans (187). AI algorithms can assist in optimizing immunotherapy regimens, including selecting the best immune checkpoint inhibitors, targeted therapies, and combination therapies. By simulating different treatment pathways and predicting treatment responses, AI can provide more precise therapeutic decisions for BTC patients (188). Furthermore, AI can integrate the analysis of the gut microbiome to further enhance personalized treatment strategies (189). Increasing evidence shows that the gut microbiome significantly influences immune therapy responses, with certain microbiome compositions enhancing immune responses and improving the efficacy of immunotherapy (190, 191). AI can analyse the microbiome data of patients to identify those whose microbiome features may affect immune therapy outcomes, thereby optimizing treatment plans (192). By integrating genomic data, immune phenotypes, clinical characteristics, and microbiome data, AI provides more comprehensive decision support for personalized treatment plans, helping to improve treatment efficacy and patient survival (193).

## 10 Future directions

As we move forward in the treatment of BTC, several key areas need further exploration and development to optimize patient outcomes. The identification of molecular subtypes in BTC, driven by specific genetic alterations such as FGFR2 fusion, IDH1 mutation, and BRAF mutation, will be essential for personalizing treatment strategies. These molecular profiles are critical for guiding targeted therapies and optimizing treatment plans. Ongoing clinical trials aim to integrate molecular profiling with precision medicine to identify patients who are most likely to benefit from specific therapies, and such efforts are expected to reshape the landscape of BTC treatment (93, 194).

The use of liquid biopsy technologies, particularly cfDNA-based assays, will play a pivotal role in the future of BTC treatment. Liquid biopsy is a noninvasive, real-time method for monitoring tumour evolution, assessing treatment responses, and detecting resistance mechanisms (42). The ability to track genetic alterations dynamically during treatment provides a powerful tool for adapting therapies and personalizing care. However, integrating these technologies into clinical practice poses challenges, including the standardization of assays, ensuring high sensitivity and specificity, and achieving clinical validation for broader use in routine diagnostics.

While monotherapies have demonstrated limited efficacy in BTC, combination therapies that involve targeted therapies, ICIs, and chemotherapy are being actively explored to improve survival outcomes. The development of reliable biomarkers to identify the patients who will benefit from these combination therapies remains a crucial research priority (110, 113, 146). The ability to accurately predict treatment response is key to enhancing patient outcomes and avoiding ineffective treatments. Additionally, the mechanisms of resistance to combination therapies need to be better understood to refine treatment protocols and prevent relapse.

Another significant development in BTC treatment is the growing use of artificial intelligence (AI) and machine learning (ML) technologies to analyse large datasets from clinical trials and patient registries. These tools are being leveraged to identify potential biomarkers, predict patient responses to different therapies, and optimize patient selection for clinical trials. AI-driven approaches are expected to improve clinical trial designs, helping to identify more appropriate patient populations and accelerating the development of new therapies for BTC (41, 195–197). However, challenges remain in integrating AI into clinical decision-making, particularly in ensuring the interpretability and clinical relevance of AI-generated insights.

## 11 Conclusion

Immunotherapy has shown great potential for treating BTC, particularly by enhancing antitumour immune responses and overcoming immune evasion mechanisms. However, the highly immunosuppressive TME in BTCs limits the efficacy of monotherapy. Combination strategies, such as immune checkpoint inhibitors with targeted therapies (e.g., FGFR2 and IDH1 inhibitors) and chemotherapy, have demonstrated promising results, significantly improving treatment outcomes. Additionally, combining immunotherapy with metabolic modulation offers new therapeutic possibilities. Although challenges such as resistance and immune-related adverse events persist, emerging therapies such as CAR-T-cell therapy, mRNA vaccines, and novel immune checkpoint inhibitors hold promise for more personalized and effective treatment options. Future research should focus on refining combination therapies, developing precise biomarkers, and conducting rigorous clinical trials to optimize immunotherapy in BTC, ultimately improving patient outcomes and quality of life.
